# Hepatitis B Virus Entry into Cells

**DOI:** 10.3390/cells9061486

**Published:** 2020-06-18

**Authors:** Charline Herrscher, Philippe Roingeard, Emmanuelle Blanchard

**Affiliations:** 1 Inserm U1259, Morphogénèse et Antigénicité du VIH et des Virus des Hépatites (MAVIVH), Université de Tours and CHRU de Tours, 37032 Tours, France; charline.herrscher@etu.univ-tours.fr; 2 Plate-Forme IBiSA des Microscopies, PPF ASB, Université de Tours and CHRU de Tours, 37032 Tours, France

**Keywords:** Hepatitis B virus, virus–host interaction, entry pathway

## Abstract

Hepatitis B virus (HBV), an enveloped partially double-stranded DNA virus, is a widespread human pathogen responsible for more than 250 million chronic infections worldwide. Current therapeutic strategies cannot eradicate HBV due to the persistence of the viral genome in a special DNA structure (covalently closed circular DNA, cccDNA). The identification of sodium taurocholate co-transporting polypeptide (NTCP) as an entry receptor for both HBV and its satellite virus hepatitis delta virus (HDV) has led to great advances in our understanding of the life cycle of HBV, including the early steps of infection in particular. However, the mechanisms of HBV internalization and the host factors involved in this uptake remain unclear. Improvements in our understanding of HBV entry would facilitate the design of new therapeutic approaches targeting this stage and preventing the de novo infection of naïve hepatocytes. In this review, we provide an overview of current knowledge about the process of HBV internalization into cells.

## 1. An Introduction to Virus Entry

Viruses are small organisms with a simple structure and composition. However, their interactions with host cells are complex and not always fully understood. Viruses are unable to promote infection through their own metabolic activities or mobility. Instead, they have evolved an ability to exploit the capacities of their hosts right from the first step in the infection process: entry into the cell.

Viruses generally bind to cell-surface proteins before interacting with specific receptors, leading to the activation of cellular signaling pathways. Some viruses, such as human immunodeficiency virus 1 (HIV-1) [1] and some herpesviruses [2], can fuse directly with the plasma membrane to gain access to the cytosol, but most viruses are dependent on endocytosis for uptake. Clathrin-mediated endocytosis and caveolin-mediated endocytosis are the best studied of the endocytosis pathways commonly hijacked by viruses. Dynamin plays a crucial role in these pathways, by pinching off endocytic vesicles from the plasma membrane [3,4]. Clathrin-mediated endocytosis is dependent on a large set of cellular proteins, including the adaptor protein AP-2, accessory proteins such as EPS15, and clathrin (reviewed in [5]). Caveolin-mediated endocytosis occurs within microdomains of the plasma membrane known as lipid rafts. These microdomains are enriched in cholesterol and sphingolipids, together with lipid-raft specific proteins: caveolins and cavins [6]. The actin cytoskeleton is essential for the maturation and trafficking of endocytic vesicles. It is also required for macropinocytosis, another endocytic pathway in which large volumes of cellular fluids are taken up in large endocytic vesicles called macropinosomes. Other pathways, grouped together as “non-clathrin non-caveolin endocytosis” have been discovered but have been less studied (reviewed in [7]).

Cell activation following binding to viral particles leads, in many cases, to the virus being internalized by the various endocytic mechanisms. Several viruses for which the entry pathways are well known are listed in Table 1. Entry into the lumen of endosomes or macropinosomes is accompanied by a change in environment, leading to changes in the viral particle resulting in the activation of the virus and its passage across the vacuolar membrane to deliver the viral genome or capsid into the cytosol.

These modifications can be triggered by exposure to low pH and by the proteolytic cleavage and activation of viral proteins [32]. Once the viruses have penetrated the cells, they arrive at their replication site, in the nucleus for DNA viruses and retroviruses, or at various sites within the cytosol for the other RNA viruses.

Virus entry into the cell and virus–host cell interactions are complex, but a detailed understanding of these aspects is essential to elucidate the mechanism of infection, to help combat existing and emerging viruses. This article reviews the current knowledge about the early events involved in hepatitis B virus (HBV) uptake into cells.

## 2. General Features of the Hepatitis B Virus

**Figure 1:**
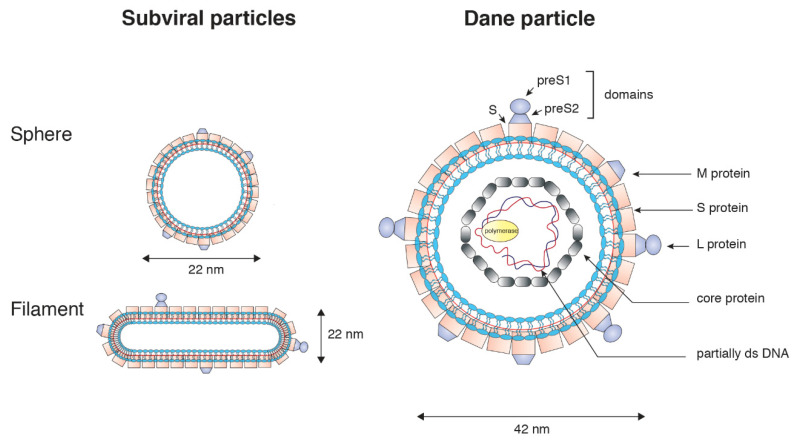
Schematic diagram of hepatitis B virus (HBV) particles.

HBV belongs to the Hepadnaviridae family. It infects exclusively hepatocytes of humans and some non-human primates. HBV is found in several different forms in the blood. The infectious form, the Dane particle, has a diameter of 42nm and contains a partially double-stranded circular DNA genome linked to a polymerase surrounded by a nucleocapsid and three envelope proteins called the large (L), middle (M), and small (S) surface proteins (Figure 1) [33]. The C-terminal S domain is common to all three envelope proteins. The M protein also contains an extra N-terminal preS2 domain, and the L protein contains a preS1 domain in addition to the preS2 and S domains [34]. The envelope proteins contain domains essential for attachment to hepatocytes.

Two other forms, secreted in large amounts and described as subviral particles (SVPs), are also present and contain only envelope proteins [35].

HBV is a small enveloped virus containing a partially double-stranded DNA genome of approximately 3.2 kilobases (Dane particle). HBV contains three envelope proteins: large (L), middle (M), and small (S). The C-terminal S domain is common to all three envelope proteins. The preS1, preS2, and S domains are indicated. The viral polymerase is covalently attached to the partially double-stranded DNA genome. The core protein forms the capsid of the viral particles. Subviral particles are non-infectious, contain only envelope proteins, and are secreted in large excess relative to infectious Dane particles.

Spherical SVPs are composed of S (90%) and M proteins (10%), whereas filamentous SVPs contain S (80%), M, and L proteins (10% each) [36]. The lipid composition of SVPs has been determined, and it appeared that phosphatidylcholine (~60%), cholesteryl ester (~14%), cholesterol (~15%), and triglycerides (~3%) are the main components [37]. Considering the relative amount of lipid representing only 25% by weight of SVPs, it has been suggested that the lipids are not organized as in a conventional membrane bilayer [38]. However, the lipid composition of Dane particles has not been determined yet. The relevance of SVPs in the life cycle remains unclear. It has been suggested that SVPs, which are secreted in much larger amounts than virions, act as a decoy for the immune system, protecting the Dane particle from the neutralizing humoral response.

The widespread use of vaccines based on HBV SVPs has significantly decreased the rate of HBV infection. However, HBV infection remains a serious threat to global health, with more than 250 million individuals estimated to be chronically infected, with a high risk of developing liver cirrhosis and hepatocellular carcinoma [39]. Current therapeutic strategies have adverse effects and low cure rates in patients with chronic HBV infection [40].

The lack of an effective treatment for the full eradication of HBV from infected hepatocytes results partly from an incomplete understanding of some of the steps in the HBV life cycle. Indeed, the major problem with this virus is the persistence of its genome in the form of a minichromosome (cccDNA). Improvements in our understanding of the various stages of its infectious cycle might facilitate the development of complementary antiviral approaches targeting these stages.

Investigations of HBV uptake were long hampered by the lack of reliable in vitro infection models. Historically, the hepatoma HepG2 cell line was used to study the last step in HBV infection, through transfection with the entire HBV genome, shedding light on many stages of the viral cycle [41,42]. However, this cell line is not susceptible to HBV infection and cannot, therefore, be used to study the mechanism of HBV entry. Primary human hepatocytes (PHHs) and the immortalized hepatic progenitor cell line HepaRG have been widely used in investigations of the early steps of viral entry [43]. However, primary human hepatocytes are highly variable between donors, the process of HepaRG cell differentiation is long, and these cells are refractory to genetic manipulation. The recent identification of sodium taurocholate co-transporting polypeptide (NTCP) as an HBV receptor has made it possible to develop hepatoma cell lines overexpressing NTCP and susceptible to HBV infection [44,45]. This easily handled model of cell infection has been used to decipher the early steps of HBV entry.

## 3. Mechanisms of HBV Entry into Cells

### 3.1. Attachment of HBV Particles to the Cell Surface

#### 3.1.1. Low-Affinity Binding to Heparin Sulfate Proteoglycans

Heparan sulfate proteoglycans (HSPGs) are glycoproteins containing one or more heparan sulfate chains found at the cell surface and in the extracellular matrix of almost all cells [46]. HSPGs have important physiological functions and have been implicated in the attachment of many viruses, including herpes simplex virus [47], human papillomavirus [48], and dengue virus [49], to cells.

**Figure 2:**
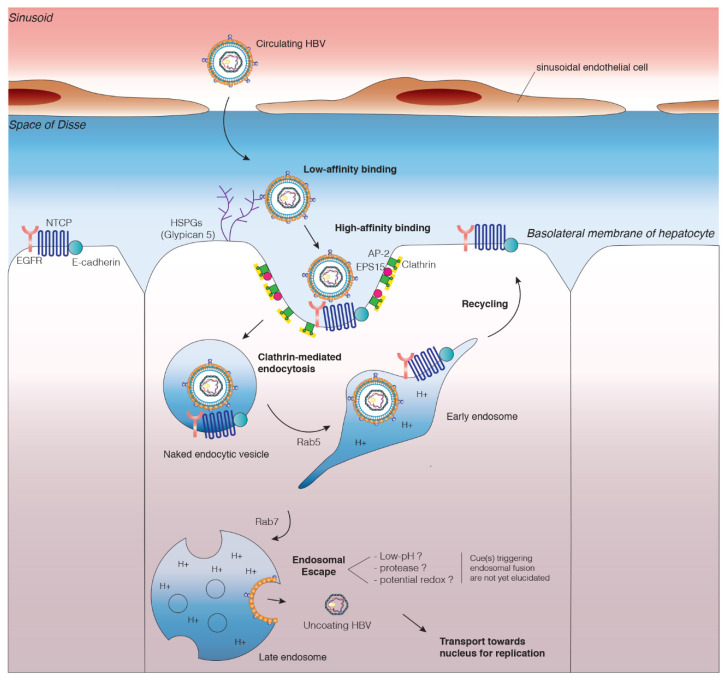
Early events in the life cycle of hepatitis B virus. HBV interacts with heparan sulfate proteoglycans (HSPGs), including glypican 5 in particular, on the hepatocyte cell membrane. HBV binds its receptor sodium taurocholate co-transporting polypeptide (NTCP) and its coreceptor epidermal growth factor receptor (EGFR). This internalization complex is associated with E-cadherin linked to N-glycosylated NTCP. This association permits the relocalization of NTCP to the plasma membrane. The HBV–NTCP–EGFR complex is taken up into the cell by clathrin-mediated endocytosis. The EGFR sorting machinery coordinates HBV transport in the endosomal network. Endosomal escape remains incompletely understood, but it has been suggested that the localization of HBV to late endosomes is crucial for productive infection and that fusion may occur in this compartment. The cues triggering endosomal fusion have yet to be clearly elucidated. Following endosomal escape, the free nucleocapsid is thought to use the microtubule network for transit to the nucleus, where it dissociates at the nuclear pore complex. Once within the nucleus, the relaxed circular DNA is converted into cccDNA, which acts as a template for transcription.

The entry of HBV into host cells has been shown to require low-affinity binding to HSPGs followed by high-affinity binding to the receptor of the virus (Figure 2). This demonstration was based on the interference of heparin, a glycosaminoglycan (GAG), with HBV attachment [50,51,52]. HBV binding to cells can be blocked more efficiently by highly sulfated inhibitors than by the less sulfated chondroitin sulfate [50,51]. This result suggests that HBV interacts only with the highly sulfated HSPGs found mostly at the surface of hepatocytes, and not with the less sulfated HSPGs present on the surface of endothelial and dermal cells [53]. Moreover, the recent observation that HBV preferentially binds glypican 5 [54], an HSPG strongly expressed in the liver, may partly account for the strong hepatotropism of HBV. However, these results do not explain how HBV avoids binding to HSPGs and becoming sequestered in other cells before it reaches the liver. HBV virions were recently shown to have two configurations differing in terms of their ability to bind HSPGs [55]. Binding to HSPGs is mediated by electrostatic interactions between the negatively charged HSPG and two positively charged residues (Arg122 and Lys141) in the antigenic loop region in the S domain present in all HBV envelope proteins [56]. This low-affinity interaction may stabilize the virus at the cell surface and promote the high-affinity binding of HBV to its receptor.

#### 3.1.2. High-Affinity Binding to the NTCP Receptor

The preS1 domain was shown early on to be essential for HBV infectivity, as it mediates the interaction of the virus with cells [57,58,59,60]. The region between amino acids 2 and 47 in preS1 has been implicated in binding to hepatocytes [61,62,63]. Recently, it has been observed that the sequence of the preS1 could modulate infectivity [64]. Indeed, it has been found that an 11 amino acid deletion in the preS1 region enhances the infectivity of HBV particles. In recent years, a number of host factors, including the asiaglycoprotein receptor, the transferrin receptor, the IL-6 receptor, and the polymerized human albumin receptor [65,66,67,68], have been proposed as possible receptors for HBV. However, none of these factors rendered hepatoma cells susceptible to HBV infection. In 2012, Yan et al. identified NTCP as a receptor for both HBV and HDV viruses [44]. This finding was independently confirmed by Ni et al., who compared the gene expression profiles of differentiated and undifferentiated HepaRG cells and demonstrated that NTCP knockdown blocked HBV infection [45]. Moreover, the exogenous expression of human or treeshrew NTCP renders hepatoma cell lines susceptible to HBV infection.

NTCP is encoded by the SLC10A1 gene. This protein is located predominantly on the basolateral membrane of hepatocytes and is responsible for the uptake into the liver of conjugated bile acids from blood [69]. NTCP is expressed in intermediate to highly differentiated HCCs, but not in poorly differentiated HCCs [70]. Its expression is downregulated in most human liver diseases. NTCP expression is rapidly lost after the isolation of primary human hepatocytes [71]. These observations may explain why malignant hepatoma cells do not support infection with HBV and HDV, and why primary hepatocytes are susceptible to HBV for only a few days after isolation.

### 3.2. Viral Entry

#### 3.2.1. Host Cell Factors Involved in HBV Internalization

After binding to the hepatocyte via NTCP, HBV must enter the cell. This entry is thought to occur via endocytosis. However, the detailed mechanisms by which NTCP mediates HBV entry remain to be determined. It also remains unclear whether HBV interacts with other receptors during cell penetration. Viruses are known to interact with several host factors to initiate entry. Hepatitis C virus interacts with at least 14 host cell factors to ensure efficient cell infection [72]. The coreceptors and host cell factors required for HBV entry have not yet been fully elucidated. NTCP is clearly necessary, but not sufficient, for effective infection. Indeed, infection efficiency remains relatively low in cell lines overexpressing NTCP [73]. These observations suggest that additional host factors are required for susceptibility to HBV infection, potentially through the formation of a complex and a multistep entry process. One recent study identified the epidermal growth factor receptor (EGFR) as a host-entry cofactor triggering HBV internalization [74]. Consistent with this finding, the HepG2 cell line has been shown to have much lower levels of EGFR than other hepatocyte cell lines [75], potentially accounting for its low rate of infection.

Another study revealed a requirement for the host cell protein E-cadherin, a calcium-dependent cell–cell adhesion protein, in HBV entry. E-cadherin has been shown to play a crucial role in HBV entry by influencing the distribution of NTCP. Indeed, this protein binds to glycosylated NTCP and facilitates NTCP relocalization to the basolateral plasma membrane [76]. Cell polarization has been described as a mandatory mechanism for the productive entry of HBV [77]. As the cell polarization induces relocalization of cell–cell adhesion proteins such as E-cadherin to the plasma membrane [78], this observation could partially be explained by the importance of E-cadherin for HBV uptake. Intriguingly, it has been established that, in contrast, cell polarization limits HCV entry through tight junctions imposing a physical barrier and restricting viral access to receptors [79,80].

#### 3.2.2. Endocytosis Mediated by Receptor Binding

Once it has interacted with its receptor and coreceptor(s), HBV must gain access to the cell. The mechanism involved in this step has become clearer since the discovery of NTCP. HBV has been shown to follow a caveolin-1-mediated entry pathway to initiate productive infection in HepaRG cells [21]. Nevertheless, a previous study reported that the treatment of primary Tupaia hepatocytes (PTH) with chemical inhibitors of caveolae-mediated endocytosis did not impair HBV infection [81].

Several studies have reported conflicting results, supporting the use of clathrin-mediated endocytosis (CME) by HBV for cell entry [16,17,18]. Indeed, the preS1 domain of HBV envelope proteins has been shown to interact with clathrin and protein adaptor 2 (AP-2) during entry into immortalized human primary hepatocytes. This result was confirmed by the significant decrease in HBV infection following silencing of the clathrin heavy chain (CHC) and AP-2 [16].

The mechanisms underlying the subsequent steps in HBV entry have only recently been studied directly in NTCP-overexpressing hepatoma cell lines, which constitute an interesting tool for deciphering HBV uptake mechanisms. A study performed in 2018 investigated HBV uptake into HepG2-NTCP cells and showed that silibinin, a drug known to inhibit clathrin-mediated endocytosis, decreased HBV entry [17]. Consistent with this result, a recent study has demonstrated the involvement of AP-2 and the EPS15 adaptor protein in the infection of HepG2-NTCP cells and PHHs with HBV [82]. Consistent with the results of these two studies, another study showed that the silencing of caveolin-1 in HepG2-NTCP cells did not decrease HBV infection, whereas the silencing of CHC, dynamin-2 (DNM2), and AP-2 resulted in a very large decrease in the level of infection. Moreover, in this study, electron microscopy analyses revealed that HBV particles were present in clathrin-coated vesicles at an early step of infection [18]. Several other lines of evidence support the notion that CME is the major route of entry for HBV in vivo (Figure 2). Indeed, CME has been shown to play a number of important roles in hepatocytes, in iron homeostasis, hepatic steatosis, and virus-induced liver infections [83]. In addition to acting as the receptor for HBV, NTCP has a physiological function in the uptake of bile salts and is recycled to the plasma membrane via CME [84].

### 3.3. Viral Escape from Endosomes

Following their endocytosis, the enveloped viruses continue along the endocytic pathway, eventually reaching the cytoplasm in early endosomes, late endosomes, or endolysosomes, depending on the compartment with the appropriate environmental cues for triggering and supporting fusion. Dependence on pH is the most important factor in the triggering of fusion for most enveloped viruses internalized by endocytosis [32]. For HBV, neither the precise site of viral fusion nor the cues triggering fusion are fully understood. Several fusogenic domains have been identified in the L protein: the C-terminal half of the preS2 region (pH-independent fusogenic domain from amino acid residues 149 to 160) [85], the N-terminal part of the S region (low pH-dependent fusogenic domain from amino acid residues 164 to 186) [86], the preS1 region (low pH-dependent) [87], and the N-terminal part of the preS1 region (low pH-dependent from amino acid residues 9 to 24) [88]. However, ammonium chloride, which raises endosomal pH, had no effect on duck hepatitis B virus (DHBV) infection, a model used in previous studies of hepadnaviral infection [89,90]. Bafilomycin A1, a potent inhibitor of vacuolar proton ATPases responsible for acidification and the establishment of a pH gradient in the endosome, inhibits trafficking from early to late endosomes [91]. The treatment of cells with bafilomycin A1 has been shown to inhibit DHBV and HBV infections [92,93]. In another study, the silencing of small GTPases involved in cargo transport between membranes, Rab5 or Rab7, led to a significant decrease in HBV infection [94]. Rab5 is responsible for transport from the plasma membrane to early endosomes, and Rab7 is responsible for transport to late endosomes and lysosomes [95].

These findings support the hypothesis that HBV is transported from early to late endosomes. Moreover, a recent study has shown that the EGFR mediates the internalization of NTCP-bound HBV via its endocytosis/sorting pathway. EGFR activation has been shown to trigger the transport of HBV to the late endosomes/lysosomes [82]. All these data strongly suggest that HBV is co-transported with EGFR and NTCP to late endosomes. However, the cues triggering fusion with the membrane remain unknown (Figure 2).

## 4. Inhibition of Entry as a Pipeline for HBV Treatment

Hepatitis B immunoglobulins (HBIGs) are currently the only “entry inhibitors” to have obtained approval for clinical use. HBIGs are polyclonal antibodies derived from pooled human plasma, directed against the AGL loop within the S domain. By binding to and neutralizing circulating virions, HBIGs prevent hepatocyte infection [96]. However, the use of HBIGs for treatment is limited by their cost and by the following additional limitations: (i) the limited supply, as these molecules are obtained exclusively from vaccinated human donors, (ii) the time-consuming nature of the purification process, and (iii) their lack of efficacy against variants of the virus with mutations of the AGL in the S domain. As a means of overcoming these limitations, monoclonal antibodies directed against the preS1 domain are currently being developed and have yielded promising results in preclinical experiments [97,98]. The application of these antibodies is currently limited to prophylactic clinical contexts, such as preventing the reinfection of liver transplants in infected patients and preventing mother–child transmission [99,100].

Blocking viral entry into naïve hepatocytes by targeting the attachment step has been explored as a possible alternative strategy (Table 2).

Natural or artificial substrates of HSPG, such as heparin, highly sulfated dextrans, suramin, and negatively charged polymers, have been reported to inhibit HBV infection [50,51,56,101,102,103]. Moreover, it has recently been demonstrated that interferon-α is able to induce soluble factors that can bind to HSPG and thus prevent viral binding [104]. This observation unravels a novel antiviral mechanism of action of interferon-α as a potent indirect-acting entry inhibitor. With the identification of NTCP as a bona fide receptor for HBV and HDV, approaches to preventing these viruses from entering hepatocytes have become a realistic and achievable option for pharmacological treatments. Moreover, a recent study suggested that de novo infection via NTCP is required to maintain the cccDNA pool [105]. Thus, the inhibition of viral entry through approaches targeting NTCP might lead to the elimination of viral cccDNA by the immune system and clearance of the infection through natural hepatocyte turnover. Several substances targeting NTCP have been tested for their ability to inhibit HBV uptake into hepatocytes. NTCP-binding molecules can be divided into two groups: (i) substrates transported by NTCP and (ii) compounds that bind NTCP without being transported (Table 2).

NTCP substrates, including taurocholate and its derivatives, were tested immediately after the discovery of NTCP as an HBV/HDV receptor. High concentrations of conjugated bile salts efficiently inhibit HBV/HDV infections [108]. However, the concentrations required to inhibit infection are well above the normal physiological range of bile salt concentrations encountered in the human body, and prolonged treatment could lead to adverse effects [109]. In addition to endogenous substrates, xenobiotics have been shown to be transported by NTCP. Indeed, ezetimibe, an inhibitor of the primary cholesterol transporter NPC1L1, is also a substrate of NTCP and has been shown to interfere with HBV/HDV entry [111]. Unlike substrate inhibitors, some peptide substrates bind to NTCP without being transported into cells. Peptides derived from HBV-preS1 and cyclosporin A (CsA) belong to this group. Myrcludex B or bulevirtide, a myristoylated 47-amino acid synthetic peptide derived from the preS1 domain of the HBV L protein is currently entering phase III clinical trials and has been shown to inhibit HBV/HDV infections efficiently [113]. CsA, a drug approved for use in immunosuppression, has antiviral activity against HBV [93]. However, inhibitors of this kind interfere with bile acid uptake, potentially leading to adverse effects. The ability of CsA derivatives to inhibit HBV entry without affecting bile acid transport has been investigated, to circumvent this problem. Two CsA derivatives, SCY450 and SCY995, have been shown to elicit strong HBV inhibition without interfering with bile acid uptake [115]. Nevertheless, detailed studies of efficacy and safety in animal models are required before these CsA derivatives can be evaluated as entry inhibitors for treatment in humans. Myrcludex B is currently the only entry inhibitor to have entered a phase III clinical trial. Myrcludex B has been shown to inhibit HBV/HDV entry at a concentration lower, by a factor of 50, than that required to inhibit bile acid uptake [119,120]. Myrcludex B can thus be used to inactivate the receptor function of NTCP at doses that do not significantly affect its natural function as a bile salt transporter, as shown by comparison to CsA.

## 5. Conclusion and Perspectives

HBV represents a threat to global health and accounts for more than 880,000 deaths each year due to complications [121]. Over 250 million people worldwide are chronic carriers of this virus.

The identification of NTCP as a specific entry receptor for HBV and HDV was a major breakthrough that has greatly advanced our understanding of HBV life cycle. HBV has been shown to bind to highly sulfated HSPGs, such as glypican 5, which is preferentially expressed in liver tissues, before interacting with its own receptor NTCP and its co-receptor EGFR. E-cadherin plays a crucial role in HBV infection, by driving NTCP to the plasma membrane. Once bound to NTCP-EGFR, HBV is internalized by clathrin-mediated endocytosis and reaches the endosomal compartment. Endosomal fusion seems to occur in late endosomes, but the cues responsible for triggering endosomal fusion have yet to be clearly identified.

HBV has been shown to produce SVPs in large excess relative to infectious particles. The purpose of the large amounts of SVPs secreted and the relevance of these particles during the life cycle of the virus remain unclear. It is widely agreed in the scientific community that SVPs probably play a role in immune system evasion. Interestingly SVPs can bind hepatocytes [63], but it is unknown whether they can then be internalized and whether these defective particles follow the same endocytic mechanism involved in HBV virion uptake. Should this turn out to be the case, it will be important to clarify the role of these particles. Deeper insight into the molecular machinery used for HBV entry should also drive the development of new HBV entry inhibitors, making it possible to eradicate HBV from infected hepatocytes.

## Figures and Tables

**Table 1:** Examples of viruses and the endocytosis pathways they use to enter cells.

Viruses	References
**Clathrin-Mediated** **Endocytosis**	
influenza A virus	[8,9]
hepatitis C virus	[10,11]
dengue virus	[12,13]
vesicular stomatitis virus hepatitis B virus *	[14,15] [16,17,18]
**Caveolae/Lipid Raft-** **Mediated Endocytosis**	
simian virus 40 hepatitis B virus *	[19,20] [21]
**Macropinocytosis**	
ebola virus	[22,23]
vaccinia virus	[24,25]
adenovirus 3	[26]
**Other pathways**	
rotavirus—IL-2 pathway	[27,28]
adenovirus 2—CLIC-GEEC pathway	[29]
coxsackievirus A9—Arf6 pathway	[30]
enterovirus 71—endophilin pathway	[31]

* Contrasting results were obtained for HBV entry.

**Table 2:** HBV entry inhibitors. Based on table from Ref. [106] and subsequently updated.

Class	Substance	Target	Status	References
Attachment inhibitors	Heparin	S, M, L	FDA-approved	[51]
	Suramin	S, M, L	FDA-approved	[103]
	Proanthocyanidin	PreS1	Preclinical	[107]
	SALP	HSPGs	Preclinical	[101]
NTCP substrate inhibitors	Taurocholic acid, UDCA, TUDCA, GUDCA	NTCP	/	[108,109,110]
	Ezetimib	NTCP/NPC1L1	FDA-approved	[111]
	Irbesartan	NTCP	FDA-approved	[112]
NTCP inhibitors	Myrcludex B	NTCP/Interferes slightly with bile acid uptake	Phase III	[113,114]
	CsA	NTCP/Interferes strongly with bile acid uptake	FDA-approved	[93]
	SCY450	NTCP/No interference with bile acid uptake	Preclinical	[115]
	SCY995	NTCP/No interference with bile acid uptake	Preclinical	[115]
	Vanitaracin A	NTCP/Interferes strongly with bile acid uptake	Preclinical	[116]
	Ro41-5253	NTCP/Antagonist of retinoic acid receptor which regulates NTCP expression	Preclinical	[117]
	Evans blue	NTCP	FDA-approved	[118]
